# A Nomogram to Predict the Risk for MACCE within 1 Year after Discharge of Patients with NVAF and HFpEF: A Multicenter Retrospective Study

**DOI:** 10.31083/j.rcm2412344

**Published:** 2023-12-12

**Authors:** Yong Wu, Yahao Zhang, Hao Jin, Jiandong Ding

**Affiliations:** ^1^Department of Cardiology, Zhongda Hospital, Southeast University, 210009 Nanjing, Jiangsu, China; ^2^Department of Geriatrics, Taizhou People’s Hospital, 225300 Taizhou, Jiangsu, China

**Keywords:** major adverse cardiovascular and cerebrovascular events, nonvalvular atrial fibrillation, heart failure with preserved ejection fraction, nomogram, prediction model

## Abstract

**Background:**

To develop and validate a nomogram prediction model for assessing the risk of major adverse cardiovascular and cerebrovascular events (MACCE) in patients with nonvalvular atrial fibrillation (NVAF) and heart failure with preserved ejection fraction (HFpEF) within one year of discharge.

**Methods:**

We enrolled 828 patients with NVAF and HFpEF from May 2017 to March 2022 in Zhongda Hospital as the training cohort, and 564 patients with NVAF and HFpEF in Taizhou People’s Hospital between August 2018 and March 2022 as the validation cohort. A total of 35 clinical features, including baseline characteristics, past medical records, and detection index, were used to create a prediction model for MACCE risk. The optimized model was verified in the validation cohort. Calibration plots, the Hosmer-Lemeshow test, and decision curve analyses (DCA) were utilized to assess the accuracy and clinical efficacy of the nomogram.

**Results:**

MACCE occurred in 23.1% of all patients within one year of discharge. The nomogram identified several independent risk factors for MACCE, including atrial fibrillation duration ≥6 years, poor medication compliance, serum creatinine level, hyperthyroidism, serum N-terminal pro-brain natriuretic peptide level, and circumferential end-diastolic stress. The DCA demonstrated the excellent efficacy of the prediction model for the MACCE end-point, with a wide range of high-risk threshold probabilities in both cohorts. The Hosmer-Lemeshow test confirmed that momogram predictions fit for both the training (*p* = 0.573) and validation (*p* = 0.628) cohorts.

**Conclusions:**

This nomogram prediction model may offer a quantitative tool for estimating the risk of MACCE in patients with NVAF and HFpEF within one year of discharge.

## 1. Introduction

Nonvalvular atrial fibrillation (NVAF) is a common heart condition that is associated with adverse outcomes in heart failure with preserved ejection fraction (HFpEF) [1, 2]. Pathogenesis, treatment, and prognosis of valvular atrial fibrillation (AF) differ significantly from NVAF [3]. For the purpose of this discussion, we will focus solely on NVAF combined with HFpEF. Patients with AF rhythm, compared to those with HFpEF in sinus rhythm, exhibit pronounced atrial dysfunction and a substantially elevated risk of cardiovascular and cerebrovascular events [4]. Persistent AF, along with other pathophysiological changes associated with the condition, may contribute to a distinct clinical AF phenotype in HFpEF; however, the optimal approach to treating and preventing AF in HFpEF is unclear. There have been many recent developments in radiofrequency ablation, anticoagulation, and rhythm control of AF [5, 6, 7]. However, their therapeutic effect on patients with HFpEF and AF remains limited, and the likelihood of cardiovascular and cerebrovascular events remains elevated in patients with HFpEF and AF compared to those without heart failure (HF) with AF. Consequently, there is a need for an individualized and practical prediction model based on the available clinical data. Nomogram fulfills this unmet need as a user-friendly prediction model to facilitate patient management and decision-making. ThereforeIn this paper we present a nomogram, the first prediction model for estimating the risk of major adverse cardiovascular and cerebrovascular events (MACCE) in patients with NVAF and HFpEF within one year of discharge.

## 2. Patients and Methods

### 2.1 Patients

**Fig. 1.**
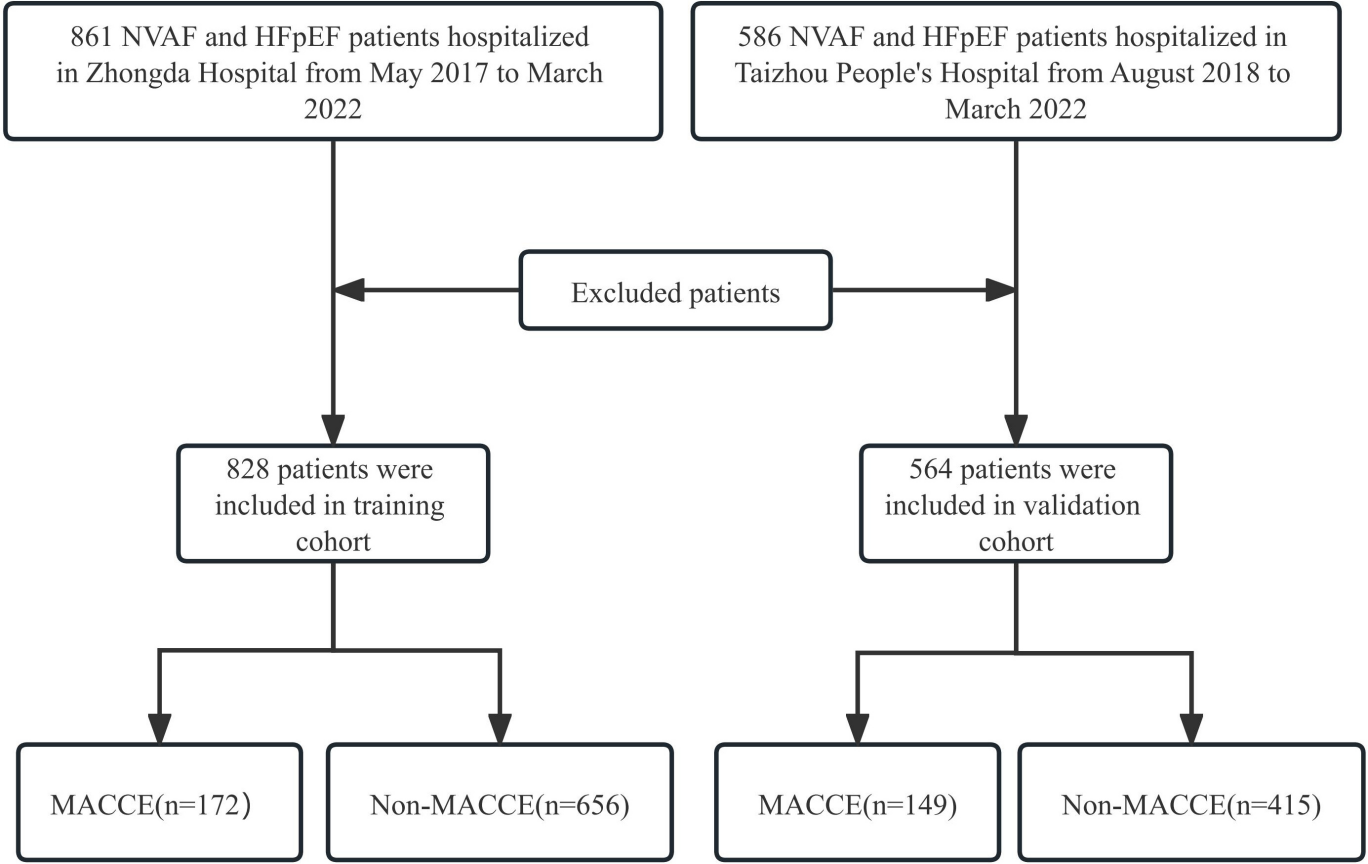
**Flowchart of patient selection**. HFpEF, heart failure with preserved ejection fraction; MACCE, major adverse cardiovascular and cerebrovascular events; NVAF, nonvalvular atrial fibrillation.

This multicenter retrospective study enrolled 861 consecutive patients with NVAF and HFpEF from May 2017 to March 2022 in Zhongda Hospital. Additionally, the validation cohort included 586 patients with NVAF and HFpEF recruited at the Taizhou People’s Hospital between August 2018 and March 2022. All participants in the MACCE group experienced cardiovascular and cerebrovascular events within one year of discharge. This study was approved by the Ethics Committee of Zhongda hospital and Taizhou people’s Hospital, respectively. Additional details can be found in Fig. 1.

The diagnostic criteria for NVAF [8] and HFpEF [9] set forth by the European Society of Cardiology were adhered to in this study. Patients meeting any of the following conditions were excluded from the study: (1) individuals with coronary artery disease who had undergone coronary artery bypass grafting or had received three or more coronary stents; (2) patients with severe congenital heart diseases such as pulmonary stenosis, large atrial septal defect, ventricular septal defect, aortic coarctation, patent ductus arteriosus, or tetralogy of Fallot; (3) individuals with severe end-stage diseases affecting vital organs, including acute or chronic liver failure, renal failure requiring blood purification, previous extensive cerebral infarction, or hemorrhage; (4) patients with malignant clonal diseases such as leukemia, lymphoma, or malignant solid tumors; and (5) individuals lost to follow-up.

### 2.2 Data Collection

The following demographic data were collected: sex, age, body mass index, smoking history, systolic blood pressure, diastolic blood pressure, classification of AF, duration of AF, ablation therapy of AF, and poor medication compliance indicated by a score of less than 6 on the score of Medication Adherence Report Scale [10]. Additionally, we collected information the administration medications including angiotensin-converting enzyme inhibitors/angiotensin receptor antagonists, beta-blockers, mineralocorticoid receptor antagonists, angiotensin-receptor neprilysin inhibitors, and sodium-glucose co-transporter-2 (SGLT-2) inhibitors. The presence of comorbidities including hyperthyroidism, hypertension, coronary artery disease, stroke, and chronic obstructive pulmonary disease, was also documented. Lab tests were performed to measure serum levels of hemoglobin, low-density lipoprotein cholesterol, total cholesterol, uric acid, serum creatinine (Scr), estimated glomerular filtration rate (eGFR), serum potassium ($K^{+}$), serum sodium (${\mathrm{Na}}^{+}$), hemoglobin A1c, and N-terminal pro-brain natriuretic peptide (NT-proBNP). Finally, left ventricular ejection fraction and left ventricular end-diastolic dimension (LVEDd) were assessed through echocardiography. The circumferential end-diastolic stress (cEDS) was calculated using the following formula [11]:

$$\mathrm{cEDS}=\frac{D\,B\,P\times {\left(\text{ LVIDs }÷2\right)}^{2}\times \left\{1+\frac{{\left(\text{ LVIDs }÷2+P\,W\,T\,s\right)}^{2}}{{\left(\text{ LVIDs }÷2+P\,W\,T\,s÷2\right)}^{2}}\right\}}{{\left(\text{ LVIDs }÷2+P\,W\,T\,s\right)}^{2}-{\left(\text{ LVIDs }÷2\right)}^{2}}$$

cEDS, the circumferential end-diastolic stress; DBP, diastolic blood pressure; LVIDs, left ventricular internal diameters; PWTs, Posterior wall thicknesses.

### 2.3 Follow-up and Outcomes

During the follow-up period, every patient was contacted via a phone call to ascertain the occurrence of MACCE. The primary outcome of follow-up was the occurrence of MACCE within one year of discharge [12], including all-cause death, nonfatal acute myocardial infarction, and nonfatal stroke.

### 2.4 Statistical Analysis

Statistical analysis was performed using SPSS 20.0 (IBM, Inc., Chicago, IL, USA) and R (version 3.6.2; R Foundation for Statistical Computing, Vienna, Austria). The mean and standard deviation of continuous variables were calculated and compared using the unpaired Student’s *t*-test or Mann-Whitney U-test while categorical variables were compared using the χ^2^ test or Fisher’s exact test. All clinical variables for both cohorts were obtained from the hospital information system. In the training cohort, univariate and multivariate analyses were conducted using the least absolute shrinkage and selection operator and logistic regression, respectively. The nomogram was established based on the results of the multivariate logistic regression via the rms package of R (version 3.6.2, R Foundation for Statistical Computing, Vienna, Austria). To achieve the optimal logistic regression, insignificant coefficients were removed from the model due to the regression penalty applied to all variables; independent variables were chosen to obtain the optimal logistic regression.

The final model to predict the occurrence of MACCE in patients with NVAF and HFpEF after discharge was developed using the rms package of R (version 3.6.2, R Foundation for Statistical Computing, Vienna, Austria). The performance of the final predictive model was evaluated by the calibration plot, Hosmer-Lemeshow test, and decision curve analysis (DCA) using the validation cohort (developed with the R packages of rms and rmda). All statistical tests were two-tailed; *p* < 0.05 was considered statistically significant.

## 3. Results

### 3.1 Participants

A total of 1392 patients were enrolled in the study, with 828 from Zhongda Hospital and 564 from Taizhou People’s Hospital. Out of these, 55 patients (33 from the training cohort and 22 from the validation cohort) were excluded from the study. There were no significant differences in the baseline data, including age, sex, body mass index, systolic blood pressure, diastolic blood pressure, hypertension, and stroke, between the MACCE and non-MACCE cohorts, except for the Scr and hemoglobin A1c levels, LVEDd, and cEDS. Detailed data analysis is shown in Table 1. The proportion of patients with MACCE in the training and validation cohorts was 20.8% and 26.4%, respectively. In the training cohort, 83 patients experienced nonfatal stroke, 37 patients experienced nonfatal myocardial infarction, and the remaining 52 patients experienced death from all-causes. In the validation cohort, 66 patients experienced nonfatal stroke, 29 patients experienced nonfatal myocardial infarction, and the remaining 54 patients experienced death from all-causes.

**Table 1.** **Patient characteristics of the training and validation cohorts**.

Characteristic		Training cohort		*p*	Validation cohort		*p*
		MACCE	Non-MACCE		MACCE	Non-MACCE	
		(n = 172)	(n = 656)		(n = 149)	(n = 415)	
Male (%)		99 (57.6)	373 (56.9)	0.87	81 (54.4)	229 (55.2)	0.86
Age, years, mean (SD)		73.51 (7.81)	74.37 (8.55)	0.23	73.45 (8.24)	72.85 (7.16)	0.40
BMI ≥28, kg/$m^{2}$, n (%)		93 (54.1)	304 (46.3)	0.07	88 (59.1)	217 (52.3)	0.16
SBP, mmHg, mean (SD)		149.2 (15.9)	147.6 (17.3)	0.27	147.6 (16.3)	146.3 (15.7)	0.39
DBP, mmHg, mean (SD)		84.4 (9.3)	85.7 (9.8)	0.12	85.9 (8.8)	86.5 (9.1)	0.49
Classification of AF				0.32			0.97
	Paroxysmal AF, n (%)	32 (18.6)	112 (17.1)		36 (24.2)	93 (22.4)	
	Persistent AF, n (%)	35 (20.3)	114 (17.4)		31 (20.8)	86 (20.7)	
	Long-term persistent AF, n (%)	66 (38.3)	235 (35.8)		42 (28.2)	123 (29.6)	
	Permanent AF, n (%)	39 (22.7)	195 (29.7)		40 (26.8)	113 (27.2)	
Duration of AF ≥6, years, n (%)		89 (51.7)	198 (30.2)	<0.01	81 (54.4)	152 (36.6)	<0.01
Ablation therapy of AF, n (%)		57 (33.1)	201 (30.6)	0.53	73 (49.0)	174 (41.9)	0.14
Smoke, n (%)		38 (22.1)	131 (20.0)	0.54	36 (24.2)	124 (29.9)	0.18
Hyperthyroidism, n (%)		41 (23.8)	53 (8.1)	<0.01	41 (27.5)	78 (18.8)	0.03
Hypertension, n (%)		124 (72.1)	505 (77.0)	0.18	112 (75.2)	282 (68.0)	0.10
CAD, n (%)		98 (57.0)	335 (48.3)	0.17	62 (41.6)	157 (37.8)	0.42
Stroke, n (%)		60 (34.9)	197 (30.0)	0.22	39 (26.2)	124 (29.9)	0.39
COPD, n (%)		25 (14.5)	78 (11.9)	0.35	13 (8.7)	45 (10.8)	0.47
Poor medication compliance, n (%)		73 (42.4)	164 (25.0)	<0.01	46 (30.9)	87 (21.0)	0.02
ACEI/ARB, n (%)		101 (58.7)	393 (59.9)	0.78	59 (39.6)	153 (35.7)	0.56
Beta-blocker, n (%)		74 (43.0)	269 (41.0)	0.63	66 (44.3)	186 (44.8)	0.91
MRA, n (%)		123 (71.5)	492 (75.0)	0.35	97 (65.1)	265 (63.9)	0.79
Antiplatelet, n (%)		139 (80.8)	511 (77.9)	0.41	115 (77.2)	311 (74.9)	0.59
DOACs, n (%)		71 (41.3)	319 (48.6)	0.09	60 (40.3)	186 (44.8)	0.34
VKAs, n (%)		29 (16.9)	77 (11.7)	0.07	21 (14.1)	54 (13.0)	0.74
ARNI, n (%)		58 (33.7)	196 (29.9)	0.33	41 (27.5)	137 (33.0)	0.22
SGLT-2 inhibitor, n (%)		48 (27.9)	268 (40.9)	<0.01	52 (34.9)	193 (46.5)	0.01
Hb, g/L, mean (SD)		115.28 (9.67)	116.42 (10.31)	0.19	117.63 (12.69)	118.32 (10.67)	0.52
LDL-C, mmol/L, mean (SD)		4.24 (1.81)	3.99 (1.78)	0.10	4.38 (1.38)	4.42 (1.54)	0.78
TC, mmol/L, mean (SD)		5.84 (2.13)	6.03 (2.25)	0.32	5.74 (1.79)	5.41 (1.88)	0.06
UA, µmol/L, mean (SD)		439.41 (122.52)	441.66 (128.9)	0.84	447.12 (115.38)	432.85 (132.08)	0.24
Scr, µmol/L, mean (SD)		147.23 (66.91)	135.45 (58.37)	0.02	153.65 (40.19)	118.55 (38.06)	<0.01
eGFR, mL/(min⋅1.73 $m^{2}$), mean (SD)		55.79 (11.35)	58.15 (14.88)	0.05	56.95 (12.12)	59.13 (13.65)	0.09
$K^{+}$, mmol/L, mean (SD)		3.89 (0.73)	3.77 (0.87)	0.10	4.04 (0.61)	3.98 (0.67)	0.34
${\mathrm{Na}}^{+}$, mmol/L, mean (SD)		129.42 (9.65)	130.06 (10.43)	0.47	131.86 (8.27)	130.35 (9.82)	0.09
HbA1c, %, mean (SD)		8.22 (2.07)	7.78 (2.14)	0.02	8.58 (1.82)	7.21 (1.74)	<0.01
NT-proBNP, pg/mL, mean (SD)		7291.5 (2381.4)	6829.6 (2127.9)	0.01	7568.1 (2154.7)	6472.6 (1848.0)	<0.01
LVEF, %, mean (SD)		61.3 (7.4)	62.6 (8.6)	0.07	63.7 (7.5)	65.1 (9.7)	0.11
LVEDd, mm, mean (SD)		48.14 (6.52)	46.86 (6.39)	0.02	48.54 (5.48)	45.05 (6.13)	<0.01
cEDS, kdyne/${\mathrm{cm}}^{2}$, mean (SD)		42.97 (9.21)	38.49 (10.63)	<0.01	43.55 (10.19)	36.43 (8.71)	<0.01

MACCE, major adverse cardiovascular and cerebrovascular events; ACEI/ARB, angiotensin converting enzyme inhibitor/angiotensin receptor antagonist; AF, atrial fibrillation; ARNI, angiotensin-receptor neprilysin inhibitor; BMI, body mass index; cEDS, circumferential end-diastolic stress; COPD, chronic obstructive pulmonary disease; DBP, diastolic blood pressure; eGFR, estimated glomerular filtration rate; Hb, hemoglobin; HbA1c, hemoglobin A1cl; $K^{+}$, serum potassium; LDL-C, low density lipoprotein cholesterol; LVEDd, left ventricular end-diastolic dimension; LVEF, left ventricular ejection fraction; MRA, mineralocorticoid receptor antagonist; ${\mathrm{Na}}^{+}$, serum sodium; NT-proBNP, N-terminal pro-brain natriuretic peptide; SBP, systolic blood pressure; Scr, serum creatinine; SGLT-2, sodium-glucose co-transporter-2; TC, total cholesterol; UA, uric acid; CAD, coronary artery disease; DOACs, direct-acting oral anticoagulants; VKAs, vitamin K antagonists; SD, standard deviation.

### 3.2 Screening of Predictors and Creating Nomogram

**Fig. 2.**
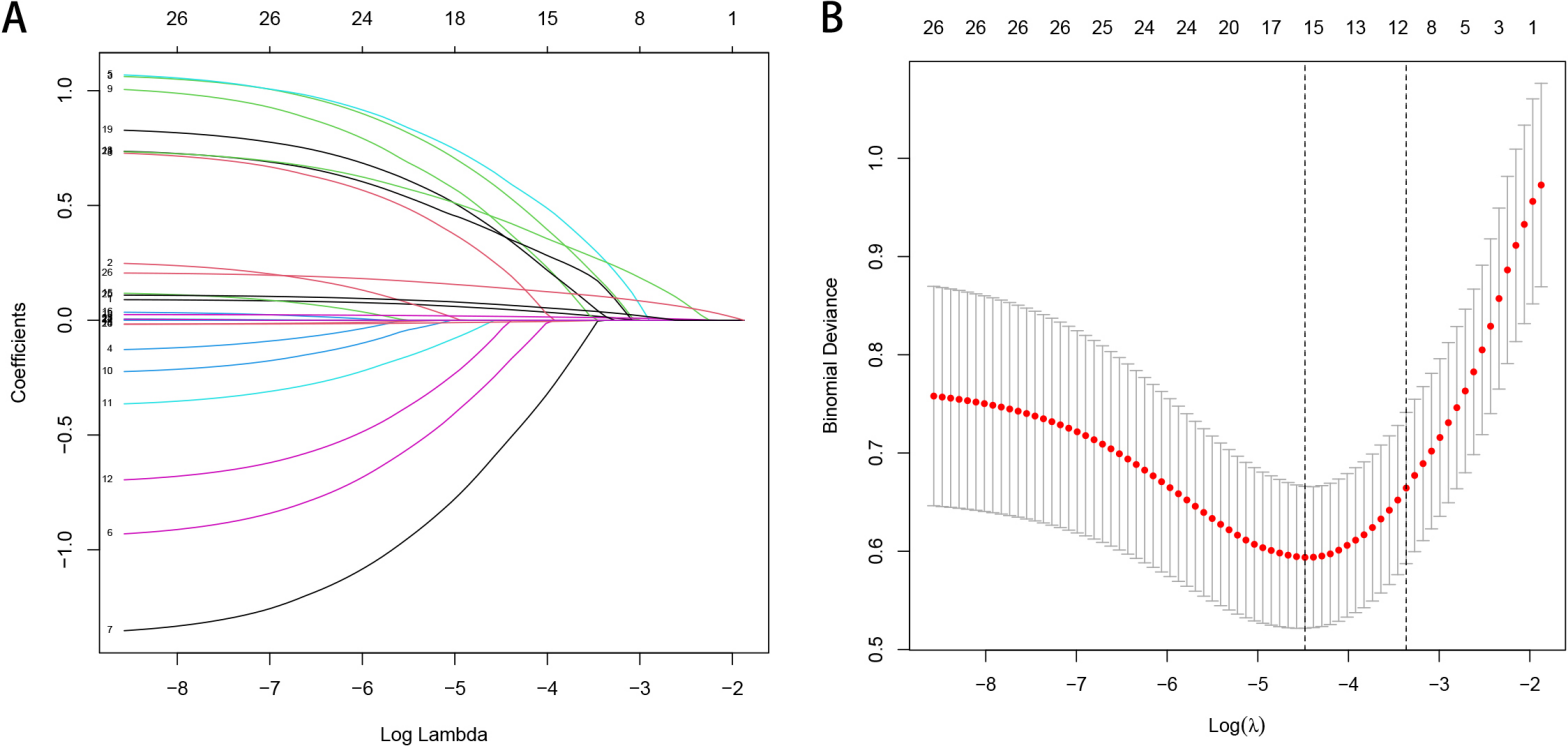
**Feature selection via the LASSO binary logistic regression model**. (A) LASSO coefficient profiles of the 24 texture features. A coefficient profile plot was produced against the log (λ) sequence. Six non-zero coefficients were sorted out by the optimal λ, when the vertical line was drawn at the value selected by 10-fold cross-validation. (B) Tuning parameter (λ) selection applied 10-fold cross-validation by minimum criteria in the LASSO model. The AUC curve was plotted vs. log (λ). Dotted vertical lines were drawn at the optimal values via the 1 standard error of the minimum criteria (the 1-SE criteria) and the minimum criteria. AUC, area under the curve; LASSO, least absolute shrinkage and selection operator; SE, standard error.

**Fig. 3.**
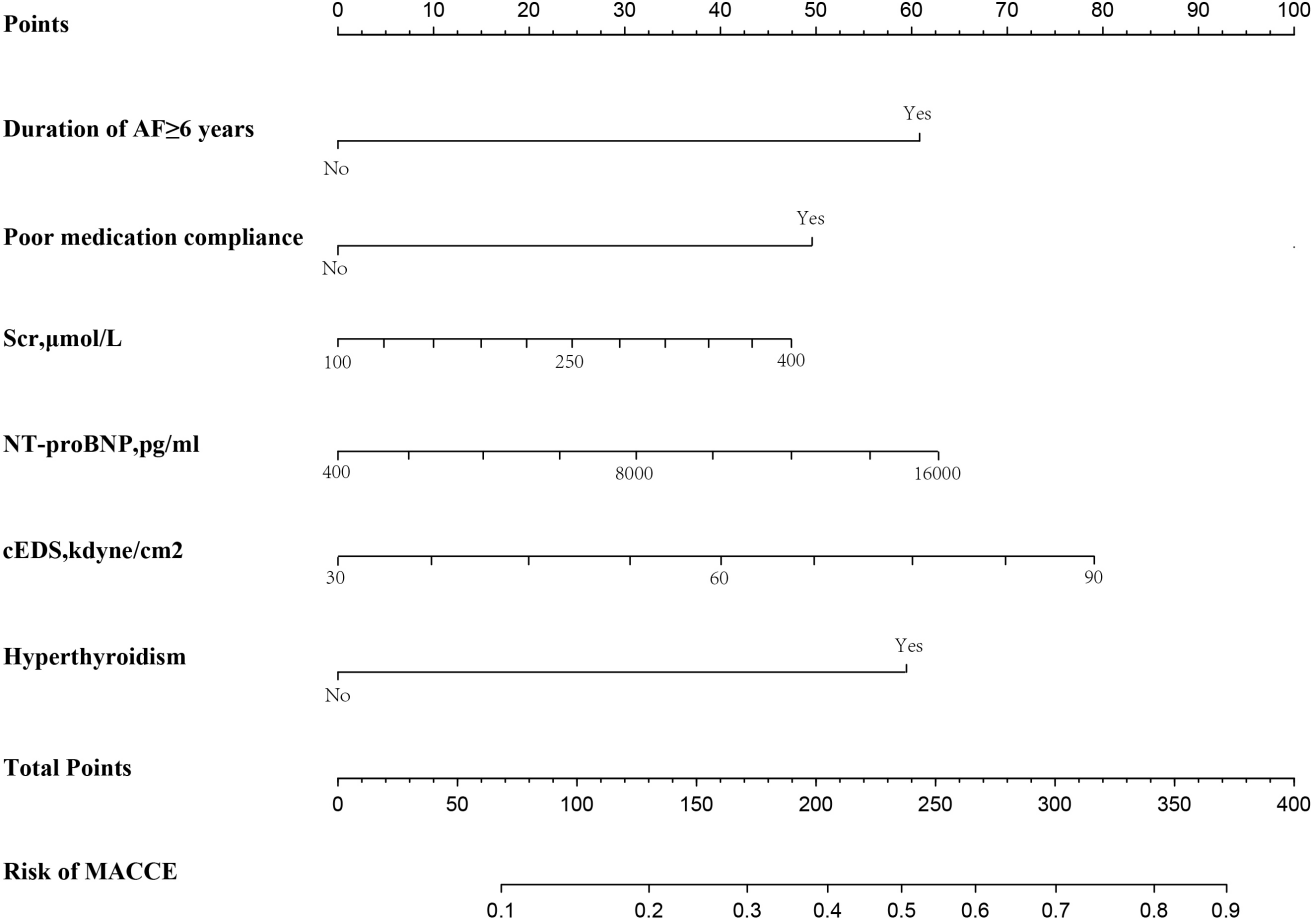
**Nomogram to predict the risk of MACCE in patients with NVAF and HFpEF within one year of discharge**. AF, atrial fibrillation; cEDS, circumferential end-diastolic stress; MACCE, major adverse cardiovascular and cerebrovascular events; NT-proBNP, N-terminal pro-brain natriuretic peptide; Scr, serum creatinine; NVAF, nonvalvular atrial fibrillation; HFpEF, heart failure with preserved ejection fraction.

The training cohort underwent least absolute shrinkage and selection operator (LASSO) regression analysis to identify predictors for the nomogram. Among the predictors screened, six variables demonstrated the strongest correlation with the occurrence of MACCE in patients with NVAF and HFpEF within one year of discharge in the training cohort (Fig. 2). These predictors included duration of AF of ≥6 years, poor medication compliance, Scr level, hyperthyroidism, serum NT-proBNP level, and cEDS. Subsequently, a multivariable logistic regression analysis confirmed the significance of these six variables in predicting MAACE risk. The odds ratio and 95% confidence intervals for each variable are shown in Table 2. Based on the probability values obtained, a nomogram was constructed (as depicted in Fig. 3) to visualize the individualized risk for MACCE using the preselected predictors.

**Table 2.** **Logistic Regression Model for Predicting MACCE**.

Variables	B	SE	Wald	*p*	OR	95% CI	
Duration of AF ≥6	1.085	0.342	10.065	0.002	2.959	2.541	3.382
Poor medication compliance	0.936	0.032	7.252	0.010	2.554	1.732	5.276
Scr	1.173	0.065	6.343	0.019	3.228	1.864	9.207
Hyperthyroidism	1.337	0.303	19.471	0.000	3.808	3.460	4.213
NT-proBNP	0.611	0.294	7.013	0.000	2.641	1.306	6.068
cEDS	0.625	0.240	6.775	0.009	1.868	1.167	2.991
Constant	0.147	0.395	4.399	0.026	1.479		

MACCE, major adverse cardiovascular and cerebrovascular events; AF, atrial fibrillation; cEDS, circumferential end-diastolic stress; CI, confidence interval; NT-proBNP, N-terminal pro-brain natriuretic peptide; OR, odds ratio, Scr, serum creatinine; SE, standard error.

### 3.3 Validation of the Prediction Model

**Fig. 4.**
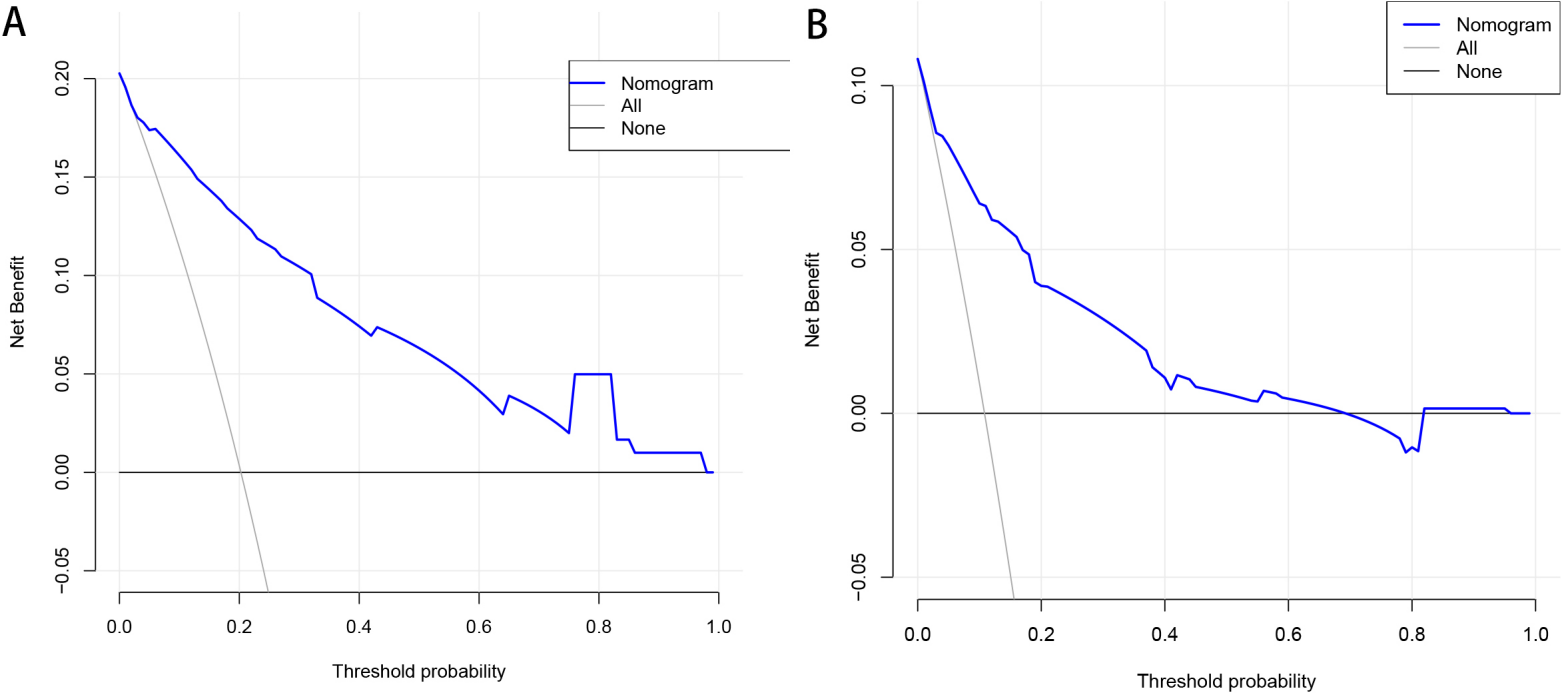
**Decision curve analysis for the training cohort (A) and the validation cohort (B)**. The horizontal line indicates that all samples are negative and the net benefit is zero. The oblique line indicates that all samples are positive. The net benefit has a negative slope.

**Fig. 5.**
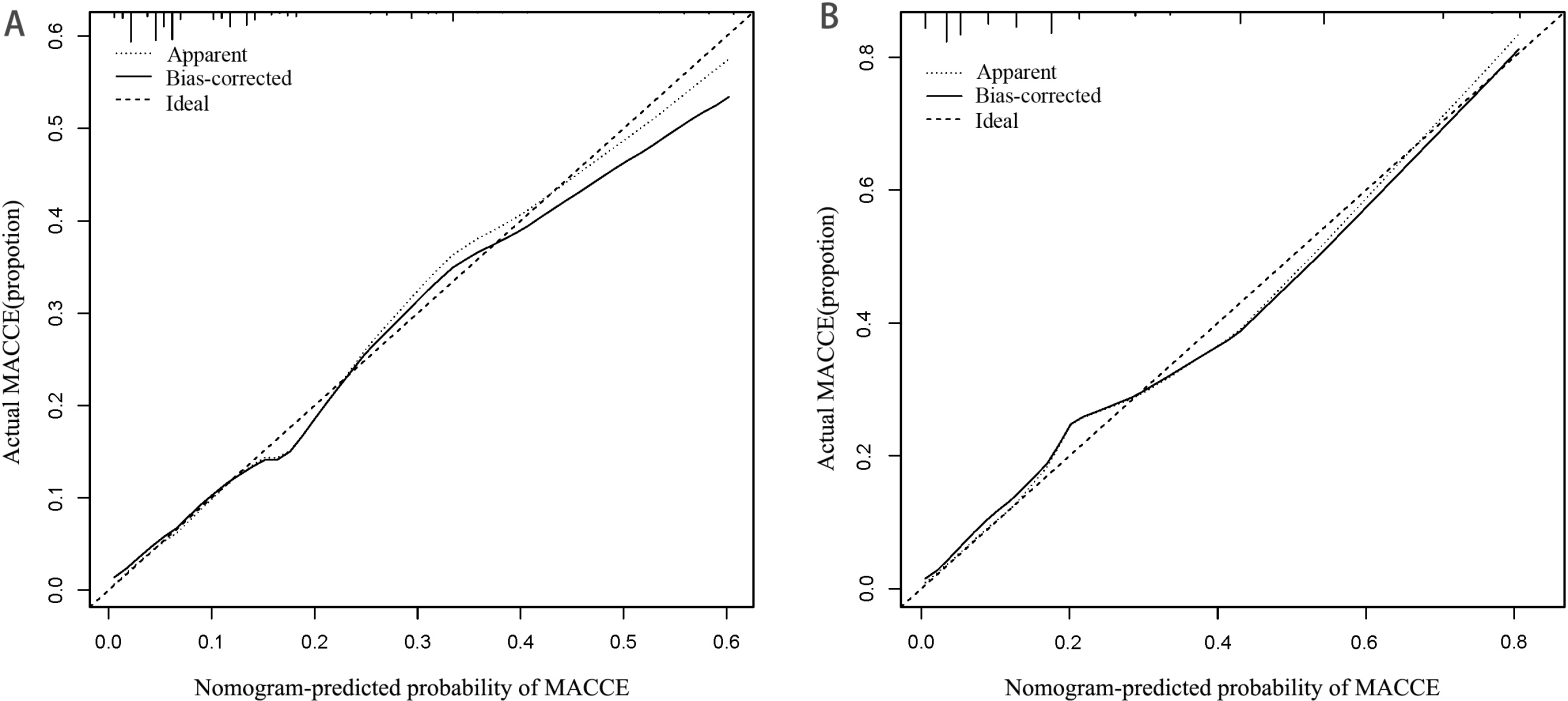
**Calibration curve of a nomogram for the training cohort (A) and the validation cohort (B)**. The X-axis represents the overall predicted probability of MACCE within one year of discharge and the Y-axis represents the actual probability. Model calibration is indicated by the degree of fitting of the curve. MACCE, major adverse cardiovascular and cerebrovascular events.

To verify the clinical validity of the nomogram, we adopted DCA in both the training and validation cohorts (Fig. 4A,B). The DCA quantified the net benefit at different threshold probabilities, indicating a wide range of high-risk threshold probabilities in both cohorts. Moreover, a calibration plot was generated to evaluate the performance of the nomogram in both cohorts (Fig. 5A,B). To further assess the goodness-of-fit, the Hosmer-Lemeshow test was conducted, yielding *p*-values of 0.573 for the training cohort and 0.628 for the validation cohort. These results indicate a high level of agreement between the observed and predicted probabilities of MACCE in both cohorts, providing additional evidence for the reliability of the nomogram.

## 4. Discussion

AF is prevalent in HFpEF and is strongly associated with adverse clinical outcomes [2, 13]. Moreover, AF burden in HFpEF is linked to a decline in left atrial distensibility [14]. These pathological changes contribute to the development of HFpEF, which is characterized by progressively enhanced ventricular interactions, worsening of pulmonary vascular disease, and right HF. A meta-analysis [15], including 14 eligible studies, demonstrated that patients with HFpEF and AF experience a poor prognosis and heavy medical burden. Therefore, establishing a prediction model is crucial for improving clinical decision-making.

In our study, we identified several independent risk factors for MACCE in patients with NVAF and HFpEF using multivariable logistic regression. These risk factors include a duration of AF of ≥6 years, poor medication compliance, Scr level, hyperthyroidism, serum NT-proBNP level, and cEDS. To assist physicians in providing appropriate clinical interventions, we developed a nomogram, which is a visual and practical tool based on these independent risk factors. Nomograms are widely used in diagnosing diseases, predicting treatment effect, and assessing clinical outcomes [16, 17, 18]. For instance, an elderly female with a 7-year history of AF (60 points) had NVAF and HFpEF, no history of hyperthyroidism (0 points), and poor medication adherence (50 points). After admission, her Scr level measured 200 µmol/L (equivalent to 18 points), serum NT-proBNP level was 4000 pg/mL (15 points), and cEDS was 68 dyne/${\mathrm{cm}}^{2}$ (55 points). Calculating her total score of 198 points, the nomogram indicates a 38% probability of experiencing cardiovascular and cerebrovascular events within one year of discharge. Therefore, we could inform this patient that the risk could be lowered to 25% as long as she took her medications on time. In summary, our nomogram provides a valuable tool for physicians to assess individual patient risks and make informed decisions for better patient management in cases of NVAF and HFpEF.

In contrast to using the receiver operator characteristic curve and the area under the curve to evaluate the model’s discriminative ability, we used DCA to validate the clinical utility of our model [19, 20]. The DCA results illustrate significant benefits in both the training and validation cohorts, indicating favorable clinical utility. Since our model is the first of its kind to predict the risk of MACCE in patients with NVAF and HFpEF, we couldn’t compare it to any existing ones. However, it is worth noting that DCA has a precedent of successful use, as it has been applied to validate Wu’s radiomics nomogram for preoperative prediction of lymph node metastasis in bladder cancer [21].

In patients with HF and normal left ventricular ejection fraction, AF significantly reduces cardiac output from baseline, leading to renal hypoperfusion and neuroendocrine changes resulting in chronic renal dysfunction. Our study revealed that elevated Scr and serum NT-proBNP levels were independent risk factors for the end-point event, suggesting an intrinsic interaction between them. Both basic [22] and clinical studies [23] establish a link between declining renal function and elevated serum NT-proBNP levels, which contribute to adverse clinical outcomes. The AKINESIS (Acute Kidney Injury N-gal Evaluation of Symptomatic heart faIlure Study) study confirmed that improved renal function is associated with mortality in acute HF, although it is not independent of hyperemia. Interestingly, achieving adequate hyperemia, as reflected by lower brain natriuretic peptide levels, was found to have a stronger associon with mortality in acute HF [24]. Hence, it is essential to remain vigilant for the possibility of renal hypoperfusion when diastolic dysfunction occurs in patients with AF.

Studies focusing on targeted metabolisms have revealed that HFpEF is associated with more severe systemic microvascular endothelial dysfunction and inflammation compared to HF with reduced ejection fraction [25]. This results in increased fibrosis in HFpEF [25]. Consequently, myocardial fibrosis leads to decreased ventricular distensibility and increased left ventricular cEDS. In our study, although significantly elevated LVEDd (*p* < 0.05) in the MACCE group did not independently impact the study outcomes during the multivariate logistic regression analysis, it indicated that cEDS elevation holds greater clinical significance than dilated LVEDd in patients with NVAF and HFpEF. Thus, researchers should place more attention to the latent damage caused by impaired cEDS over cardiac systolic function [26, 27].

SGLT-2 inhibitors demonstrated a significant reduction in the risk of MACCE in our study population (*p* < 0.05), regardless of the presence of diabetes mellitus. A multicenter randomized trial [28] highlighted that dapagliflozin treatment significantly improved patient-reported symptoms, physical limitations, and tolerance in patients with chronic HFpEF within 12 weeks. Similarly, a multinational randomized trial showed substantial clinical benefits of empagliflozin without harm in patients hospitalized with acute HF within 90 days of treatment initiation [29].

However, in this study, the impact of SGLT-2 inhibitors in patients with HFpEF and AF may be diminished when considering confounding factors, especially medication adherence. Additionally, the TOPCAT (Treatment of Preserved Cardiac Function Heart Failure with an Aldosterone Antagonist Trial) [30] analysis of three phenotypes in HFpEF revealed varying effects of spironolactone treatment. In the OPTIMIZE-HF (Organized Program to Initiate Lifesaving Treatment in Hospitalized Patients with Heart Failure) [31] study, digoxin treatment in elderly patients with HF (regardless of HF with reduced ejection fraction or HFpEF) reduced the risk of readmission without significantly affecting mortality. Hence, it is essential to recognize that no single drug can independently influence a patient’s endpoint events.

Results from the ARISTOTLE (Apixaban for Reduction in Stroke and Other Thromboembolic Events in Atrial Fibrillation) trial [32] indicate that thyroid disease, regardless of classification, does not affect the clinical outcomes in patients with AF. However, the combination of AF and hyperthyroidism led to significantly worse outcomes. Our study focused on NVAF, where HF was a comorbidity, and our patients had multiple underlying illnesses. In recent years, the ${\mathrm{CHA}}_{2}$${\mathrm{DS}}_{2}$-VASc score has been used for mortality risk stratification. A ${\mathrm{CHA}}_{2}$${\mathrm{DS}}_{2}$-VASc score ≥5 predicts an increased risk of all-cause mortality and readmissions for all causes, regardless of AF status, in patients with HF over 75 years [33]. Therefore, a comparison of our model with ${\mathrm{CHA}}_{2}$${\mathrm{DS}}_{2}$-VASc should be an essential component of a comprehensive evaluation.

Our nomogram offers a precise estimation of the risk of MACCE within one year of discharge, incorporating six easily obtainable early in the admission process. Our model has undergone external validation, confirming its effectiveness and accuracy. As a result, the findings of our study could enhance patient adherence to treatment and follow-up, enabling them to better understand the disease risk and make informed decisions.

## 5. Limitations

Our study has some limitations. (1) This was a retrospective analysis study and although we included patients from two centers, the sample size was not big enough; (2) The high proportion of people lost to follow-up might have led to bias; (3) Considering that the coronavirus disease 2019 epidemic might have an impact on our study results, we need to revalidate the model after the disease is completely controlled; (4) To make it easier for clinicians to use our model, an app for assessment needs to be developed and continuously improved.

## 6. Conclusions

The novel nomogram prediction model developed in this study to predict the risk of MACCE in patients with NVAF and HFpEF within 1 year after discharge based on six independent predictors, namely duration of AF of ≥6 years, poor medication compliance, Scr level, hyperthyroidism, serum NT-proBNP level, and cEDS demonstrated good discriminative power and calibration.

## Data Availability

The datasets used and/or analyzed during the current study available from the corresponding author on reasonable request.
